# The Seasonal Dietary Shift and Niche Resilience of Yaks on the Qinghai–Tibetan Plateau

**DOI:** 10.3390/ani16040613

**Published:** 2026-02-14

**Authors:** Shuai Zheng, Yuning Ru, Mengyuan Xu, Yushou Ma, Yuan Ma, Na Guo

**Affiliations:** 1 Qinghai Provincial Key Laboratory of Adaptive Management on Alpine Grassland, Academy of Animal Science and Veterinary Medicine, Qinghai University, Xining 810016, China; 2 School of Life Sciences, Shandong University, Qingdao 266237, China

**Keywords:** foraging strategy, dietary selection, niche breadth, yak, Qinghai–Tibetan Plateau

## Abstract

Understanding how herbivores adjust their foraging strategies to cope with seasonal resource fluctuations has been central to the nutritional ecology. Optimal Foraging Theory (OFT) predicts that generalists should broaden their dietary niche when high-quality resources are scarce, but empirical evidence in extreme environments remains poorly understood. We used *trn*L-P6 metabarcoding of fecal samples (*n* = 10/season) and a local reference library of 120 plant species to quantify diet composition and niche metrics of free-ranging yaks (*Bos grunniens*) on the Qinghai–Tibetan Plateau in June (summer) and October (autumn) 2024. Yaks shifted from a diverse, forb-dominated diet (e.g., Polygonaceae, Rosaceae) in summer to a specialized diet dominated by grasses in autumn. Although dietary richness and total niche width (TNW) decreased in autumn, phylogenetic diversity remained stable, indicating a strategic shift to distinct evolutionary lineages to ensure functional redundancy. Furthermore, food network analyses demonstrated a transformation from a flexible, modular foraging pattern in summer to a highly integrated, synchronized network in autumn. These findings suggest that under the distinct quality–quantity trade-off of high-altitude ecosystems, yaks adopt an energy-maximization strategy by minimizing search costs, aligning with the opportunity cost constraints of OFT, rather than randomly expanding their niche. This insight into selective foraging dynamics is critical for developing sustainable grazing practices that accommodate the natural adaptive behaviors of alpine herbivores.

## Simple Summary

Ecological theory posits that seasonal food availability drives dramatic temporal variation in dietary niche breadth, yet alternative paradigms offer contrasting predictions about its effect. Traditional Optimal Foraging Theory predicts that herbivores should broaden their dietary niche when resources become scarce. However, it remains unclear how high-altitude specialists like the yak adjust their fine-scale foraging strategies to navigate the extreme transition from resource-rich summers to resource-limited autumns. By sequencing plant DNA from fecal samples, we analyzed the seasonal diet dynamics of grazing yaks on the Qinghai–Tibetan Plateau. We found a dramatic dietary shift, as instead of expanding their niche under autumn scarcity, yaks significantly contracted a dietary niche, switching from diverse high-quality forbs to specialized abundant graminoids. These results suggest that yaks function as adaptive mixed feeders, employing a selective energy-maximization strategy to persist in one of the world’s harshest environments.

## 1. Introduction

Understanding the foraging strategies of large herbivores is fundamental to ecology, as food resources are often the primary limiting factor for ungulate populations [1,2]. While the physiological functions of nutrient intake, such as body maintenance, thermoregulation, and reproduction, are well established [3], the specific drivers behind food choice remain complex. Herbivores do not simply consume available biomass; they must navigate a landscape filled with heterogeneous plant species, varying phenological states that influence forage palatability [4,5]. This challenge is particularly acute in seasonal environments, where animals must adapt to drastic fluctuations in forage properties and availability. Consequently, how herbivores balance their dietary needs against these environmental constraints directly dictates their body condition, survival, and ultimately, population dynamics [6,7].

Optimal Foraging Theory (OFT) and niche theory provide contrasting frameworks for predicting how herbivores adjust their dietary niche width in response to seasonal resource fluctuations. The classic “prey model” of OFT posits that diet breadth is inversely related to the availability of high-quality resources; as preferred foods become depleted during lean seasons, foragers are predicted to broaden their diet to include lower-quality items [8,9]. Conversely, niche-based models suggest that resource scarcity intensifies competition, forcing individuals or species to specialize on narrower subsets of resources to minimize overlap, whereas abundant resources during “fat seasons” allow for dietary generalization and overlap [10,11]. Consistent with niche-based models, meta-analyses have found that dietary partitioning was generally greatest when food abundance was low, suggesting that competition for limited food drives partitioning [12]. Despite these theoretical advances, empirical evidence remains conflicting [13,14]. Recent research on ungulates (e.g., bushbucks) in heterogeneous landscapes implies that these adjustments are more nuanced which is driven by energetic trade-offs, herbivores may narrow their realized niches to maximize energy intake under fluctuating conditions [15]. However, it remains unknown foraging strategies for ruminant prioritize in extreme environments where resource transitions are rapid and severe.

Tibetan Plateau, with a mean elevation exceeding 4000 m above sea level, is the world’s largest and highest alpine grassland [16]. It represents one of the world’s harshest grazing environments, characterized by an exceptionally short plant growing season of only 90 to 120 days [17]. Despite these environmental challenges, the plateau supports unique biodiversity. The yak (*Bos grunniens*) is an iconic symbol of high-altitude, providing the essential resources, including meat, milk, and fuel for millions of nomadic pastoralists in high-altitude [18,19]. For these large herbivores, the growing season is critical for survival, and previous studies have emphasized the general impact of seasonality on alpine herbivores [20]; the fine-scale adaptive strategies occurring during the phenological transition from summer to autumn remains poorly understood. Unlike the growing season in summer, autumn presents a unique quality–quantity trade-off, while food biomass remains relatively high, nutritional quality declines rapidly due to plant senescence [21]. This transitional period is decisive, as yaks must maximize energy intake to accumulate sufficient reserves for the upcoming non-growing season. Therefore, assessing whether yaks adopt a niche-expansion strategy or maintain selective segregation in response to these autumnal resource fluctuations is critical for understanding their survival mechanisms, with potential applications in resource management and environmental monitoring.

Traditional methods in determining herbivores diet include direct observation, stomach or fecal microbiology, infrared spectrophotometry, and stable isotope analyses [22]. These methods are effort-intensive and often lack the resolution to construct the fine-scale diet composition and the subtle patterns of co-occurrence among plant items, for grazing yaks, which are highly mobile and feed on diverse plant species [23,24,25,26]. Compared to the traditional methods, DNA metabarcoding may reveal more detailed information of herbivores’ diet and offers an opportunity to reconstruct herbivore diets webs [1,20].

Therefore, we quantified the seasonal dynamics of dietary niches and food web of grazing yaks in summer and autumn using DNA metabarcoding. We hypothesized that (1) yak diet composition and food web exhibit seasonal turnover, and (2) the seasonal shifts drive an expansion of niche width or niche differentiation. Results from this study could provide insights into the behavioral mechanisms yaks employ to navigate the trade-off between resource quality and quantity.

## 2. Materials and Methods

### 2.1. Study Site

The study was conducted in Huangyuan County (3100 m a.s.l.; 36.9133° N, 100.9346° E), located on the Qinghai–Tibetan Plateau. The region is characterized by a typical continental monsoon climate, with a mean annual temperature of −0.4 °C (ranging from −11.9 to 11.7 °C) and mean annual precipitation of 505 mm in 2024. Approximately 80% of the rainfall occurs during the plant growing season from May to October. The dominant vegetation is alpine meadow. The dominant and associated species included *Elymus nutans*, *Festuca rubra*, *Poa crymophila*, *Stipa aliena*, *Carex przewalskii*, *Kobresia humilis*, *Saussurea superba*, *Oxytropis kansuensis*, *Ajania tenuifolia*, *Potentilla nivea*, *Tibetia himalaica*.

### 2.2. Sample Collection

Fecal samples were used as a non-invasive proxy to determine the diet of free-ranging adult yaks. To ensure a representative sampling, samples were collected from different herds grazing within the study area. Sampling was conducted in June (summer, growing season, *n* = 10) and October (autumn, senescent season, *n* = 10) in 2024. Fecal samples were collected immediately after observing different individuals defecate, with each defecation event sampled only once, using sterile spatulas to minimize ground contamination, placed into sterile tubes, and flash-frozen in liquid nitrogen. All samples were transported to the laboratory and stored at −80 °C until DNA extraction.

### 2.3. Local DNA Reference Library Construction

To ensure accurate taxonomic identification of dietary plants, we constructed a local DNA reference library. A comprehensive botanical survey was conducted across the study area, and voucher specimens of all encountered plant species (*n* = 120) were collected. Plant identification was confirmed by expert botanists [27]. For each species, total genomic DNA was extracted from silica-dried leaf material. The *trn*L (UAA) intron P6 loop region was amplified using the c/d primers [28] and sequenced via the Sanger sequencing. All curated sequences were compiled into a local, high-quality reference database for subsequent taxonomic assignment. These associated DNA data were archived in the National Library of Medicine (NCBI).

### 2.4. DNA Extraction, Amplification, and Sequencing

Total genomic DNA was extracted from 0.2 g of each fecal sample using the QIAamp^®^ Fast DNA Stool Mini Kit (Qiagen, Valencia, CA, USA), following the manufacturer’s protocol [1,20]. Extraction blanks were included throughout the process to monitor for potential contamination. DNA concentration and quality were assessed using a NanoDrop 2000 spectrophotometer (Thermo Scientific, Wilmington, DE, USA) and 1.5% agarose gel electrophoresis, respectively [1]. The P6 loop of the chloroplast trnL (UAA) intron was amplified using the primers *trn*L-g (5′-GGGCAATCCTGAGCCAA-3′) and *trn*L-h (5′-CCATTGAGTCTCTGCACCTATC-3′) [28]. PCR amplicons were purified, pooled, and sequenced on an Illumina NovaSeq 6000 platform, generating 2 × 300 bp paired-end reads.

### 2.5. Bioinformatic and Statistical Analyses

The raw sequence data was processed using the OBITools4 pipeline [29]. Reads were assigned to their respective samples using *ngsfilter*. Low-quality sequences (mean quality score <30) and those with ambiguous nucleotides were discarded. Identical reads were dereplicated into unique sequences using *obiuniq*. A stringent filtering process was then applied using *obiclean* to remove putative PCR and sequencing errors, excluding variants with low abundance (≤1000 reads total), short length (≤10 bp), or those differing by only one nucleotide from a much more abundant sequence (≤0.05%). Sequences with low identity (≤95%) to our local reference database were also removed. Taxonomic assignment of the final, high-quality molecular operational taxonomic units (mOTUs) was performed against local reference library with a 100% identity criterion. The relative read abundance (RRA) of each plant taxon was used to quantify dietary composition. To standardize sequencing depth, the mOTU table was rarefied to 2000 reads per sample. This process was repeated 10 times, and the averaged RRA values were used for all subsequent ecological analyses to ensure robustness [1,30].

Dietary diversity was assessed using Hill numbers of order q = 0 (species richness) and q = 1 (the exponential of the Shannon index), calculated with the *hilldiv* v1.5.3 R package. Dietary phylogenetic diversity was calculated using the *picante* v1.8.2 package. A phylogenetic tree of identified plant taxa was constructed by aligning the *trn*L sequences with *MAFFT* v7.525 and inferring the tree with *FastTree* v2.1.11 [31,32]. We calculated the Mean Phylogenetic Distance (MPD) to evaluate the phylogenetic breadth of the diet. The diet-plant network was visualized using a bipartite graph with the *bipartite* v2.23 R package [33].

We constructed a Bray–Curtis dissimilarity matrix based on the dietary mOTU data and performed a non-metric multidimensional scaling (NMDS) analysis using the *vegan* v2.7.2 package in R. Pairwise differences in dietary composition between seasons were tested using PERMANOVA [34]. We constructed and visualized plant co-occurrence networks to explore potential associations among dietary components. Based on the relative abundance matrix, pairwise Spearman correlations were calculated using the *Hmisc* v5.2.3 package. Significant correlations (|r| > 0.6, *p* < 0.05) were retained to build undirected networks with the *igraph* v 2.1.4 package, and the networks were visualized using *ggraph* v2.2.2 [35,36,37].

## 3. Results

### 3.1. Seasonal Dietary Diversity and Composition

**Figure 1:**
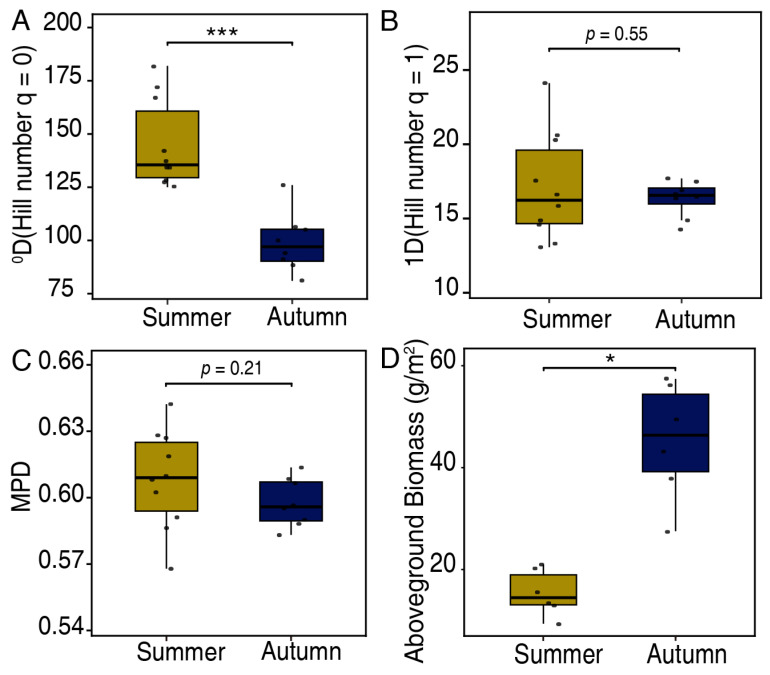
Dietary diversity of yaks and above-ground biomass of alpine grassland vegetation in both summer and autumn. (**A**) dietary richness (0D). (**B**) dietary richness and evenness (1D). (**C**) mean pairwise phylogenetic distance (MPD, overall clustering of food plant taxa on the phylogenetic tree). (**D**) aboveground biomass of the study area where yak foraging. Statistical significance was assessed using the Wilcoxon test (* *p* < 0.05, *** *p* < 0.0001).

Total 20 samples included 510 unique trnL-P6 sequences (mOTUs) from 44 plant families. The diet dietary of yaks exhibited a dramatic seasonal shift between summer and autumn. Dietary richness (Hill number q = 0) was significantly higher in summer than in autumn (144.8 ± 1.5 vs. 109 ± 2.0, *p* < 0.05), dietary diversity (q = 1) and phylogenetic diversity (MPD) had no significant difference between summer and autumn (Figure 1A–C), while aboveground biomass of alpine grassland was higher in autumn than in summer (Figure 1D).

**Figure 2:**
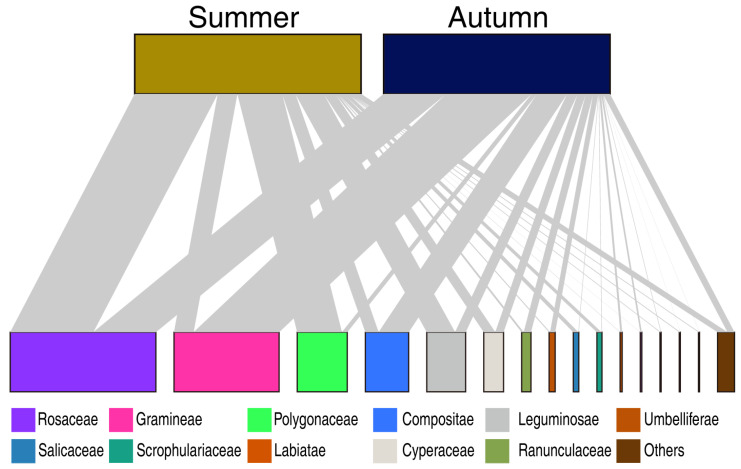
Dietary composition of yak in both summer and autumn. Bipartite networks depicting the dietary composition of yaks. Lines connect yaks (**upper bar**) to dietary plant sequences (**lower bar**), which are colored by plant family. The width of the upper bar represents the overall dietary composition, the links represent the proportional contribution of each plant family, and the width of the lower bar represents the total intake proportion of each plant family.

**Figure 3:**
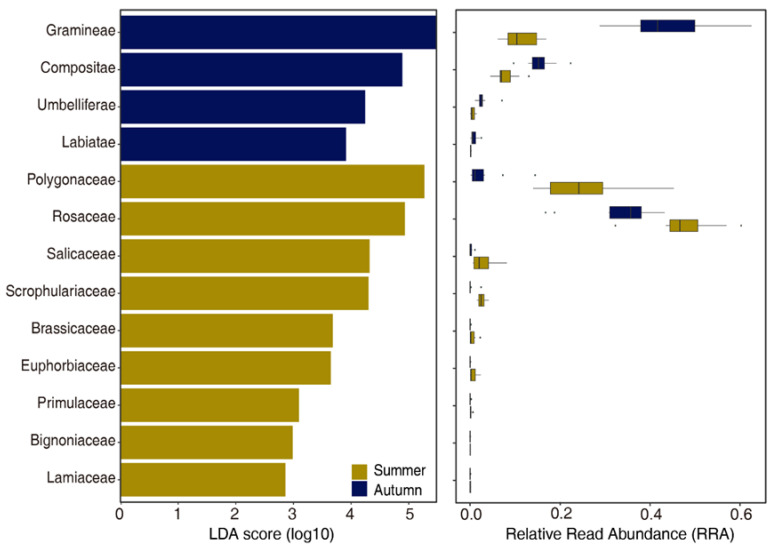
Key plant taxa between month and its relative read abundance (RRA). The diagram in the left was the results of LEfSe analysis on dietary data of yak in two seasons, showing the linear discriminant analysis (LDA) scores (threshold = 2) of food components consumed by yaks in different seasons, along with boxplots of the relative read abundance (RRA) for the corresponding plant families.

Examination of quantitative food-web interactions revealed seasonal shifts regarding main dietary plant families between summer and autumn. At the family level, the top 10 plant taxa in terms of relative read abundance (RRA) across both seasons included Rosaceae, Polygonaceae, Fabaceae, Poaceae, Cyperaceae, Asteraceae, Caryophyllaceae, Ranunculaceae, Liliaceae, and Primulaceae in both summer and autumn. The diet composition in summer was dominated by forbs, with Rosaceae (36%), Polygonaceae (21%), and Fabaceae (12%) being the most abundant families. In contrast, mean RRA of Poaceae (grasses) was greatest (39%), less for Asteraceae (13%), lesser for Cyperaceae (3.6%) and Polygonaceae (2.4%) in autumn (Figure 2). To determine which plant taxa most contributed to the dietary niche between summer and winter in yaks, LEfSe analysis was performed. Rosaceae and Polygonaceae were the significant diet biomarkers in summer, whereas Poaceae and Apiaceae characterized in autumn (Figure 3). Overall, the yaks transitioned from a forb-dominated diet in summer to grass dominated diet in autumn.

### 3.2. Seasonal Food Plant Partitioning

**Figure 4:**
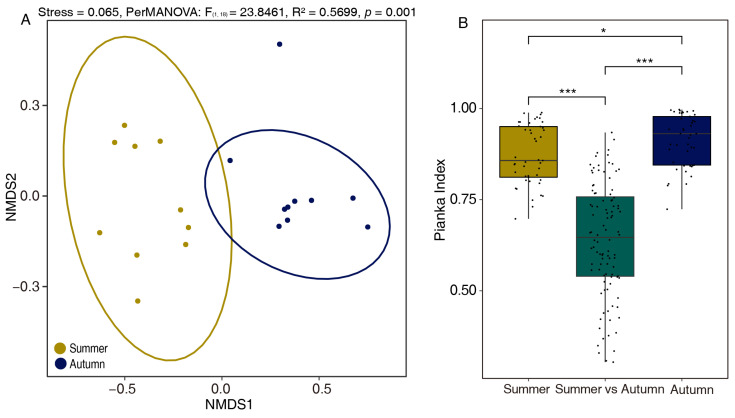
Seasonal variation in yak dietary composition. (**A**) Non-metric multidimensional scaling (NMDS) analysis based on Bray–Curtis similarity of dietary data. (**B**) Pianka index values representing dietary niche overlap among months (* *p* < 0.05, *** *p* < 0.0001); values closer to 1 indicate higher similarity, while values closer to 0 indicate lower overlap. The colors represent paired Pianka index values within/among months.

**Figure 5:**
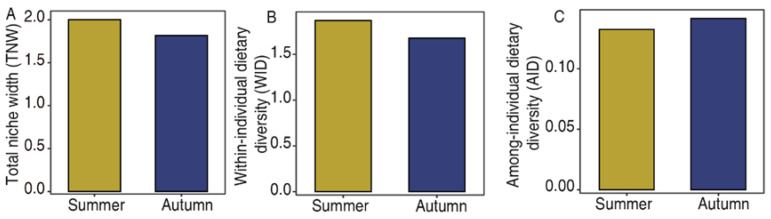
Variation of niche breadth in yaks between summer and autumn. (**A**) Total Niche Width (TNW, reflecting the overall niche width). (**B**) Within Individual Diversity (WID, reflecting the proportion of individual niche width in the total niche width of the population). (**C**) Among Individual Diversity (AID, reflecting the proportion of among-individual differences in total niche breadth).

Nonmetric multidimensional scaling (NMDS) based on dietary dissimilarity revealed clear seasonal partitioning of plant taxa consumed between summer and autumn among individual fecal samples (PERMANOVA: F = 23.85, R^2^ = 0.57, *p* = 0.001) (Figure 4A). These results are consistent with the pairwise Pianka niche-overlap index (Figure 4B), which showed a mean value of 0.8730 ± 0.0122 in summer compared to 0.9135 ± 0.0107 in autumn. The total niche breadth (TNW) was significantly higher in summer than in autumn (1.87 vs. 1.67, *p* = 0.034) (Figure 5A), consistent with the higher dietary species richness observed. Within-individual diversity (WID) was lower in autumn than in summer (Figure 5B), whereas among-individual diversity (AID) showed the opposite trend, increasing in autumn (Figure 5C). Together with the Pianka index results, the higher AID/TNW ratio (degree of specialization) in autumn indicates that as food resource quantity and quality declined, individual differentiation became more pronounced despite the increase in overall dietary overlap.

### 3.3. Food-Webs Roles of Plant Taxa

**Figure 6:**
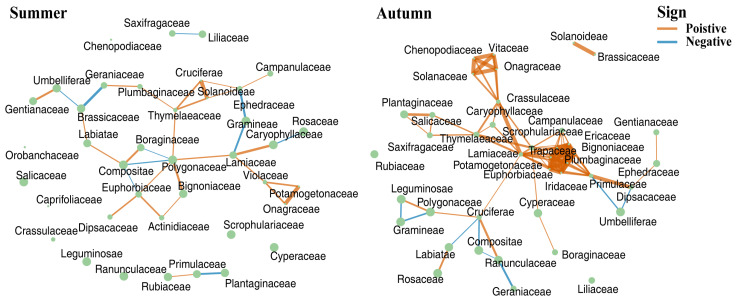
Co-occurrence network of dietary components in the yaks. Each node represents a distinct plant taxon detected in the yak diet. Node size indicates the relative read abundance of the corresponding plant family (nonlinear scaling). Edge width reflects the strength of the co-occurrence relationship, and edge color denotes the sign of the correlation (positive or negative).

The co-occurrence networks of dietary plant composition revealed a distinct seasonal shift in foraging strategy between summer and autumn (Figure 6). The dietary network in summer was sparser and more modular, characterized by fewer edges, higher modularity and a higher proportion of positive correlations. Although Rosaceae was the most abundant family, Polygonaceae served as a key hub species, connecting different dietary modules. This modular pattern indicates that yaks evolved a more opportunistic and flexible foraging strategy, switching between different patches of preferred forbs, rather than consuming resources in a synchronized manner. Food web in autumn was denser and more integrated, exhibiting increased edge density, a higher clustering coefficient, and greater assortativity. It was characterized by a large, interconnected core of co-consumed plants, suggesting that yaks consistently consume a core set of available grasses and forbs together. Notably, a negative correlation between Poaceae and Fabaceae within food network indicates avoidance foraging pattern.

## 4. Discussion

Although seasonal dietary plasticity is well-documented in generalist herbivores, empirical investigation into how population-level niche dynamics emerge from individual foraging strategies during critical phenological transitions remains limited. This lack of knowledge is particularly critical for alpine ungulates, which are compelled to optimize their foraging strategies amidst the severe seasonal trade-off between declining forage quality and abundant standing biomass. By leveraging the high taxonomic resolution of DNA metabarcoding, we demonstrate that free-grazing yaks exhibit a fundamental reconfiguration of their dietary niche between summer and autumn. We show that yaks transition from a diverse, forb-dominated diet (e.g., Rosaceae and Polygonaceae) in summer to a specialized, grass- and sedge-dominated diet (e.g., Poaceae) in autumn. This shift is driven by two complementary mechanisms: a contraction of the total niche breadth (TNW) coupled with an unexpected increase in individual specialization (AID/TNW) and network connectivity. Our results suggest that yaks adopt a context-dependent energy-maximization strategy, with narrowing their niche to focus on abundant and high-fiber resources, while maintaining distinct individual foraging patterns to mitigate competition. These findings suggest a potentially generalizable framework for understanding how the realized dietary niches of alpine herbivores are constrained by the dynamic interplay of seasonal phenology and physiological plasticity.

### 4.1. Seasonal Dietary Diversity and Niche Breadth

The results reveal a distinct seasonal pattern in dietary richness and diversity driven by the phenological trade-off between resource quality and quantity. Although aboveground biomass was found to be higher in autumn than in summer, likely due to the accumulation of lignified graminoid stems, yaks displayed a contraction in their dietary niche in autumn. Specifically, dietary taxonomic richness peaked in summer, whereas autumn was characterized by narrowing niche, despite phylogenetic diversity showing no significant difference. This seasonal contraction can be interpreted through the framework of OFT, particularly regarding opportunity costs [8,38]. In the resource-rich summer, the abundance of high-quality vegetation allows yaks to be selective, foraging on a wide richness of plants. However, in autumn, as diverse forbs senesce and become scarce, the opportunity cost of searching for these rare, nutrient-dense items becomes prohibitively high. Consequently, yaks minimize search effort by shifting their focus to the most reliable and abundant food sources such as grasses. Although the metabolic energy of grasses declines in autumn, their high biomass makes them the most economical energy source in helps of gut microbiome [21,39]. This strategy supports the theoretical prediction that animals facing higher starvation risk or search costs will prioritize intake over nutritional quality [40], resulting in the observed reduction in dietary richness and niche breadth in autumn.

Variations in dietary profiles often reflect underlying disparities in nutritional quality, driven by factors such as individual physiological requirements and environmental heterogeneity [41]. Although direct nutritional assessment was not conducted in the present study, our previous investigation into the annual nutritional dynamics of alpine forage [20], together with established ecological principles suggest that summer diets provide peak nutrition, whereas high-quality resources become progressively depleted in winter [42]. In this study, yaks prioritized ephemeral, nutrient-dense resources in summer. This foraging behavior closely parallels that of the North American bison, which similarly concentrates on high-quality graminoids and forbs during the productive season to meet the metabolic demands of a large body size [43,44]. Crucially, by exploiting fast-growing vegetation in summer, herbivores can accumulate somatic energy reserves while preserving persistent forage stocks for the winter range. This mechanism, often termed the ‘storage effect’, serves to mitigate seasonal resource bottlenecks and alleviate intraspecific competition [45,46]. These findings underscore the morphophysiological plasticity of large herbivores in response to environmental variability [10]. Deciphering the limits of this flexibility is vital for adaptive management in the face of rapid climate change [47,48], where scalable, data-driven approaches are essential for revealing previously obscured patterns of dietary adaptation in wildlife populations.

### 4.2. Seasonal Shifts in Diet Composition

Yaks diet vary seasonally, and available evidence suggests that our results are likely to conservatively describe dietary separation. In summer, yaks heavily utilized nutritious forbs, particularly from the Polygonaceae and Rosaceae families (e.g., *Polygonum sibiricum*, *P. viviparum*, *Potentilla anserina*). These species green up quickly and provide high protein content, facilitating rapid compensatory growth and fat accumulation before winter. However, the consumption of these diverse forbs reflects a nutritional strategy, yaks prioritize digestible energy and protein over the metabolic cost of detoxification. Many of these plants are rich in plant secondary metabolites (PSMs) [21,49,50,51,52]. The ability to tolerate these compounds is likely attributed to the yak’s symbiotic gut microbiota, which functions as a ‘biological shield’ by metabolizing potentially harmful PSMs (e.g., tannins and alkaloids) into non-toxic compounds [53]. This co-evolved capacity enables yaks to exploit nutrient-dense resources in the growing season that would otherwise be unpalatable to less adapted herbivores. In autumn, grasses and sedges became most common in yak diet, which are common throughout alpine grassland and provide lower-quality resources in autumn [21] that yaks switch to when higher-quality resources are most limited. The seasonal shifts in diet composition further highlight remarkable physiological adaptations to the quality–quantity trade-off. As ruminants indigenous to the Qinghai–Tibetan Plateau, yaks harbor a specialized and highly efficient gut microbiome capable of degrading complex structural carbohydrates (i.e., cellulose and hemicellulose) [54]. These microbial communities ferment high-fiber substrates into volatile fatty acids (VFAs), serving as the primary energy source for the host [55,56]. This enhanced fermentation capacity allows yaks to maximize nutrient extraction from abundant fiber-rich plants, thereby mitigating nutritional limitations and maintaining high intake rates to support fat reserves before the onset of the harsh winter.

Large herbivorous mammals are typically categorized along a functional continuum, ranging from grazers that specialize on monocots (mainly grasses) to browsers that consume dicots, with mixed feeders consuming substantial amounts of both [57,58]. While yaks are commonly described as grazers within traditional foraging guild, our result suggests mixed-feeding strategies may be common in high-altitude large herbivores. This dietary plasticity reflects an adaptive response to specific biogeographical variables of the regions, particularly the intense seasonal fluctuations and winter snow cover [59]. Consistent with recent insights from Yellowstone ecosystem [60], our data indicate that in the extreme environments, extrinsic environmental forcing often exerts a stronger influence on diet. Consequently, strict dietary specialization may be less common than previously assumed in highly seasonal habitats. Instead, dietary generalization serves as a dominant survival strategy, allowing herbivores to navigate the complex spatiotemporal dynamics of plant phenology and nutritional availability [61].

### 4.3. Seasonal Dietary Niche Separation and Food Webs

Seasonality clearly influenced the predominance of cluster assignments for both the plants and animals in food network. Ecologists have long debated the drivers of population niche width, contrasting the Niche Variation Hypothesis (NVH), which posits that population niche expansion stems from individual specialization [10], with Optimal Foraging Theory (OFT), which assumes conspecifics broaden their diets uniformly under resource limitation [8]. Typically, both theories predict niche expansion under scarcity. Unlike previous findings in moose (Alces alces), which tend to maintain a generalist strategy or expand their niche under scarcity [46], food scarcity did not drive niche expansion but rather contraction in yaks. The divergent trends observed in TNW and AID between seasons suggest a distinct foraging strategy. In summer, the high TNW and modular network structure reflect an opportunistic generalist strategy consistent with OFT in resource-rich environments However, the autumn dynamics present a fascinating deviation. While TNW contracted due to resource senescence, the increase in AID suggests that individuals differentiated their foraging paths within this constrained niche. This aligns with the NVH’s premise that intraspecific competition drives individual specialization, likely mediated by searching and handling disparate food items (e.g., scarce forbs vs. abundant grasses).

This individual partitioning is mirrored in the transformation of the food co-occurrence network. The modular structure observed in summer reflects a flexible foraging strategy where yaks exploit various high-quality patches without strong coupling. In contrast, the dense, integrated network in autumn suggests a constrained synchronization, where the population is forced to converge on a core set of grasses and sedges. Crucially, the negative correlation between Poaceae and Fabaceae in the autumn network highlights a stark functional trade-off. As suggested by foraging theory [62], individuals are constrained by cognition or physiology from maximizing intake of all food types simultaneously. In autumn, yaks focus on maximizing biomass intake from abundant but low-quality grasses and sedges and may lack the time or foraging efficiency to seek out rare, high-protein legumes, and vice versa. This trade-off drives the observed niche partitioning, preventing complete dietary overlap even when resources are scarce.

These findings have implications for grassland management. Since the autumn diet shifts towards fibrous grasses and sedges, the primary nutritional bottleneck becomes protein limitation [63,64]. Therefore, management interventions may shift from generic supplementation to strategic nitrogen delivery during the phenological transition. Providing catalytic protein sources (e.g., urea-molasses blocks) specifically in autumn would maintain microbial fermentation efficiency, allowing yaks to better utilize the abundant dry grass biomass and minimize body weight loss during the cold season.

Low sample size may not sufficiently uncover the full range of food items that yaks consumed. The dominant dietary components and main feeding interactions in the communities were most likely captured, allowing robust characterization of broad seasonal shifts in yak dietary niches and food-web structure. Although the dietary niche of yaks exhibited clear shifts between summer and autumn, the limited temporal sampling restricts inference about fine-scale transition dynamics, such as early versus late summer or early versus late autumn dietary variation. Further incorporating larger sample sizes and finer temporal sampling across the Qinghai–Tibetan Plateau will refine our understanding of yak foraging dynamics and trophic interactions.

## 5. Conclusions

This study characterizes the seasonal diet niche and network dynamic of yaks on the Qinghai–Tibetan Plateau. We observed that yaks shifted from a forb-dominated diet in summer to a diet primarily composed of grasses and sedges in autumn. Instead of expanding their dietary niche in response to reduced diet resources in the autumn, yaks appeared to narrow their niche while maintain stable dietary phylogenetic diversity. Yaks exhibited consistent foraging patterns, characterized by distinct plant co-occurrence networks. These findings provide insights into the dietary plasticity of large herbivores in alpine grassland and highlight the importance of aligning grazing management with plant phenology.

## Data Availability

The raw data supporting the conclusions of this article will be made available by the authors on request.
